# Genetic Influences Are Virtually Absent for Trust

**DOI:** 10.1371/journal.pone.0093880

**Published:** 2014-04-07

**Authors:** Paul A. M. Van Lange, Anna A. E. Vinkhuyzen, Danielle Posthuma

**Affiliations:** 1 Department of Social and Organizational Psychology, VU University Amsterdam, Amsterdam, The Netherlands; 2 Department of Complex Trait Genetics, Department of Functional Genomics, Center for Neurogenomics and Cognitive Research (CNCR), Neuroscience Campus Amsterdam, VU University Amsterdam, Amsterdam, The Netherlands; 3 The University of Queensland, Queensland Brain Institute (QBI), St. Lucia, Australia; 4 Department of Clinical Genetics, VU University Medical Center, Amsterdam, The Netherlands; 5 Department of Child and Adolescent Psychiatry, Erasmus University Rotterdam, Sophia Child Hospital, Rotterdam, The Netherlands; Macquarie University, Australia

## Abstract

Over the past decades, numerous twin studies have revealed moderate to high heritability estimates for individual differences in a wide range of human traits, including cognitive ability, psychiatric disorders, and personality traits. Even factors that are generally believed to be environmental in nature have been shown to be under genetic control, albeit modest. Is such heritability also present in social traits that are conceptualized as causes and consequences of social interactions or in other ways strongly shaped by behavior of other people? Here we examine a population-based sample of 1,012 twins and relatives. We show that the genetic influence on generalized trust in other people (trust-in-others: *h^2^* = 5%, ns), and beliefs regarding other people’s trust in the self (trust-in-self: *h^2^* = 13%, ns), is virtually absent. As test-retest reliability for both scales were found to be moderate or high (*r* = .76 and *r* = .53, respectively) in an independent sample, we conclude that all variance in trust is likely to be accounted for by non-shared environmental influences. We show that, relative to cognitive abilities, psychiatric disorders, and classic personality variables, genetic influences are smaller for trust, and propose that experiences with or observations of the behavior of other people shape trust more strongly than other traits.

## Introduction

Over a decade ago, “all human behavioral traits are heritable” was stated as the first law of behavior genetics [1]. While provocative at the time, evidence since then has accumulated to suggest heritability estimates of 30% or higher on assessments of cognitive ability, a variety of psychiatric disorders, and even for most classic personality traits [2]–[4]. Indeed, a few years later, one may even add a qualifier to the first law “All human behavior traits are *quite* heritable” (italics added). But the question is whether the quantifier “*all*” is justified. Is all human behavior quite heritable? Or are there exceptions to this law?.

Most behavior genetics studies have examined traits which primary causes are located within the person, either because they are linked to differences in skill or ability (such as intelligence), because they are linked to psychiatric disorders (such as schizophrenia, autism, or ADHD) or because they are part of the self, such as the classic personality variables [2]. But what about individual differences traits that are not directly linked to ability, disorders, or classic personality traits. In particular, what about individual differences that are closely related to beliefs we hold about other people? The present research seeks to illuminate the magnitude of the genetic influences on generalized trust that people have in others (trust-in-others), and beliefs about the trust that others have in themselves (trust-in-self).

We focus on trust, because we assume that trust should be strongly linked to own social interaction experiences, as well as to public information conveyed in the various media that might affect beliefs and feelings relevant to trust. Granted, there are other traits as well that presumably are strongly linked to social interactions and media influences, such as cooperative motivation or agreeableness. However, as we will outline, we assume that beliefs and feelings relevant to trust are strongly rooted in the others’ behavior. Compared to trust, traits such as cooperative motivation and agreeableness may be more strongly rooted in “the self”.

Trust is often defined as “the intention to accept vulnerability based upon the positive expectations of the intentions or behavior of another” [5]. As such, trust involves vulnerability, or uncertainty and risk that comes with the control another person has over one’s outcomes, and “positive expectations” which often imply a set of beliefs in the cooperative intentions or behavior of another person, or other people more generally [6]. Decades of research have revealed a remarkable variation among individuals in their basic levels of trust. Some people readily accept vulnerability and have positive expectations of other people, while others do not. Relative to those with low trust, high trust individuals are more likely to behave cooperatively in the face of uncertainty and conflicting interests [7]–[9], report greater life satisfaction, exhibit greater physical health, and live longer [10]. Such evidence is often explained in theories that emphasize social interaction experiences as a powerful determinant of the development of individual differences in trust [11]–[12].

Extending research and theory, we suggest that social interaction experiences may shape not only the trust we have in others (trust-in-others) but also the beliefs we hold about other people as to their trust in ourselves (trust-in-self). For example, when we repeatedly are involved in cooperative interaction, we may develop trust in others, and – perhaps more implicitly – the belief that others trust us. Alternatively, non-cooperative interaction experiences may undermine trust in others, as well as beliefs that other people trust us. Hence, both forms of trust might be considered as key examples of traits that are closely linked to experiences or observations of social interactions that are strongly affected by the others’ behavior.

This claim has received considerable support in the literature on human cooperation and social dilemmas, in which individuals face conflicts between self-interest and collective interests [13]. But also in the other contexts, others’ behavior can exert important influences on trust. For example, there is recent research showing that person’s trust in others in general might be promoted when another person takes good care of that person when in a somewhat vulnerable situation (e.g., when attaching electrodes for measuring heart rate) [14]. Also, more indirect sources of social information, such as information about human behavior in the media, might also affect our basic trust in others (and perhaps, to some degree, others people’s trust in ourselves). Thus, our major thesis is that trust-in-others and trust-in-self should reveal low heritability as it is strongly shaped by direct experience with or observations of the behavior of other people.

Here we examine the causes of individual variation in “trust-in-others” and “trust-in-self”, using scales that were validated in a large sample that is representative of the Dutch adult population. The scales are summarized in Table 1, and their psychometric properties are discussed in the Materials and Methods. In examining the genetic influences on trust-in-others and trust-in self, we adopt an extended family twin design using a Dutch population based sample (n = 1,012) of 186 identical and 191 non-identical twins, 157 non-twin siblings, and 146 parents, 151 spouses, and 181 children of the twins and siblings. In doing so, we examine the degree to which individual variation in trust is linked to genetic factors (additive and non-additive) and environmental factors (shared and non-shared between siblings). Compared to the classic twin design, the extended twin design has at least three desirable qualities: It has relatively more power, it enables us to provide more precise and less biased estimates of genetic and environmental influences, and it allows modeling of non random mating of spouses and gene-environment correlation [15]–[16].

**Table 1:** Scales for Measuring Trust-in-Others and Trust-in-Self.

The following statements are about your impression of “most other others in your environment”. These can be friends, acquaintances, colleagues, or unknown others as long as you face them every now and then - that they are part of your environment. We ask you for each of the following statements to indicate the degree to which agree or disagree with the statement.
*1 = Completely disagree*
*2 = Largely disagree*
*3 = Slightly disagree*
*4 = Agree nor Disagree*
*5 = Slightly agree*
*6 = Largely agree*
*7 = Completely agree*
**Trust-in-Others**
1. I dare to put my fate in the hands of most other people
2. I completely trust most other people
3. When push comes to shove, I do not trust most other people (r)
**Trust-in-Self**
1. I think that most other people dare to put their fate in my hands
2. I think that most other people trust me
3. When push comes to shove, most other people do not trust me (r)

Note: The headings “trust-in-others” and “trust-in-self” were not used in the actual questionnaire. They are included here for reasons of clarity. (r) indicates reverse-scored.

## Materials and Methods

### Ethics Statement

The data were collected as part of a large ongoing project on the genetics of cognition [17]–[18] for which we obtained (a) from the participants written consent for information to be stored and used for research, and (b) ethical approval by the Medical Ethical Testing Committee (METC) at the VU University Amsterdam. The analyses are based on data of adults aged 18 years or older.

### Sample and Procedure

Data included 1,012 twins and relatives (451 men, 561 women; 44.6% and 55.4%) from 264 different families who at the time of participation were registered at the Netherlands Twin Register [19]. The mean number of participating family members was 3.8. The average age of the participants at the time of measurement was 45.3 years (*SD* = 14.10, range: 17–70). The sample included 377 twins (49.3% MZ) and 157 siblings, and 146 parents, 151 spouses, and 181 children of the twins and siblings.

Determination of zygosity of same-sex twins was based on DNA polymorphisms (89 pairs, 83.96%) or, if information on DNA markers was not available, on questions about physical similarity and confusion of the twins by family members and strangers. Agreement between zygosity diagnoses from survey and DNA was 97% [20]. All five zygosity groups were reasonably well represented: monozygotic males (MZM: N = 235; 23.2%), monozygotic females (MZF: N = 256; 25.3%), dizygotic males (DZM: N = 115; 11.4%), dizygotic females (DZF: N = 237, 23.5%) and dizygotic opposite sex (DOS: N = 169; 16.7%).

### Measurement and Reliability

The trust-in-others and trust-in-self scales were designed to include three items that were central in existing scales [5]–[8], thereby capturing items with positive valence (“I completely trust most other people”) and negative valence (“When push comes to shove, I do not trust most other people”), both of which explicitly used the word “trust”, and an item that captured the broad behavioral implication of the trust: the intention to accept vulnerability, as explicated in one of the most widely-accepted definitions of trust [5] (“I dare to put my fate in the hands of most other people”). The three-item scales of trust-in-others and trust-in-self were pre-tested in 2006 in an online survey administered by TNS/NIPO, a Netherlands Institute for Public Opinion. In this pretest, using a large sample (*n* = 1804; 849 men, 955 women; age range 18–98, *M* = 46.63, *SD* = 16.53) the scales for trust-in-others (α = .68) and trust-in-self (α = .77) were deemed reliable, especially in light of the fact that both scales comprised only three items, and included 2 positive items and 1 negative item. To provide evidence for the temporal stability, we examined the test-retest reliability in an independent sample of 59 participants (44 women, 15 men; age range 18–71, *M* = 40.54, *SD* = 15.187) over a period of two months. Test-retest reliability of the trust-in-others and the trust-in-self scales were high or moderate: *r* = .76 and *r* = .53, respectively, and provide an upper limit to the estimate of heritability. The internal consistency and test-retest reliability were judged as suitable, and hence, the scales were included in the present project. At the same time, we acknowledge that the relatively modest sample size, along with the moderate test-retest reliability for trust-in-self, allow us to provide preliminary (rather than conclusive) evidence for the heritability of trust, especially for trust-in-self.

We calculated mean sum scores for trust-in-others and trust-in-self in our genetics of cognition samples of 1012 twins and relatives. As in the pretest, the trust-in-others and trust-in-self scales were reliable (respective αs = .69, and .73). All measures were corrected for age and sex to avoid spuriously increased similarities in MZ and same-sex DZ twin pairs. For all analyses, 40 (3.8%) individuals were excluded due to missing values on the trust-in-others scale and 26 (2.8%) individuals were excluded due to missing values on the trust-in-self scale. The complete questionnaire was sent out to participants by mail, and yielded a response rate of 76%.

## Results

The mean sum scores of the trust-in-others and trust-in-self scales were 15.88 (*SD* = 2.78) and,13.48 (*SD* = 3.69) respectively. The two constructs were correlated (*r* = .558, p<.001) and thus shared about 31 percent of the variance. As noted earlier, the scales are conceptually different, in that trust-in-others focuses on (generalized) trust in others, whereas trust in self focuses on beliefs regarding others’ trust in the self (see Table 1). The fact that they share variance may be partially explained by the notion that social interactions often involve either mutual trust or mutual distrust, which people develop cooperative interaction or noncooperative interactions, respectively. Thus, from an interaction perspective, trust-in-others and trust-in-self are likely to be interrelated.

On average, men and women did not significantly differ in terms of trust-in-others (unstandardized *β* = .46, *SE* = .24, *t* = 1.97, *p* = .051) or trust-in-self (unstandardized *β* = .26, *SE* = .18, *t* = 1.50, *p* = .134). The only effect we observed was a significant association with age, such that younger people were more likely to have greater trust-in-others (unstandardized *β* = −.03, *SE* = .01, *t* =  −3.25, p<.01) and greater trust-in-self (unstandardized *β* = −.01, *SE* = .01, *t* =  −2.17, *p*<.05).

**Figure 1:**
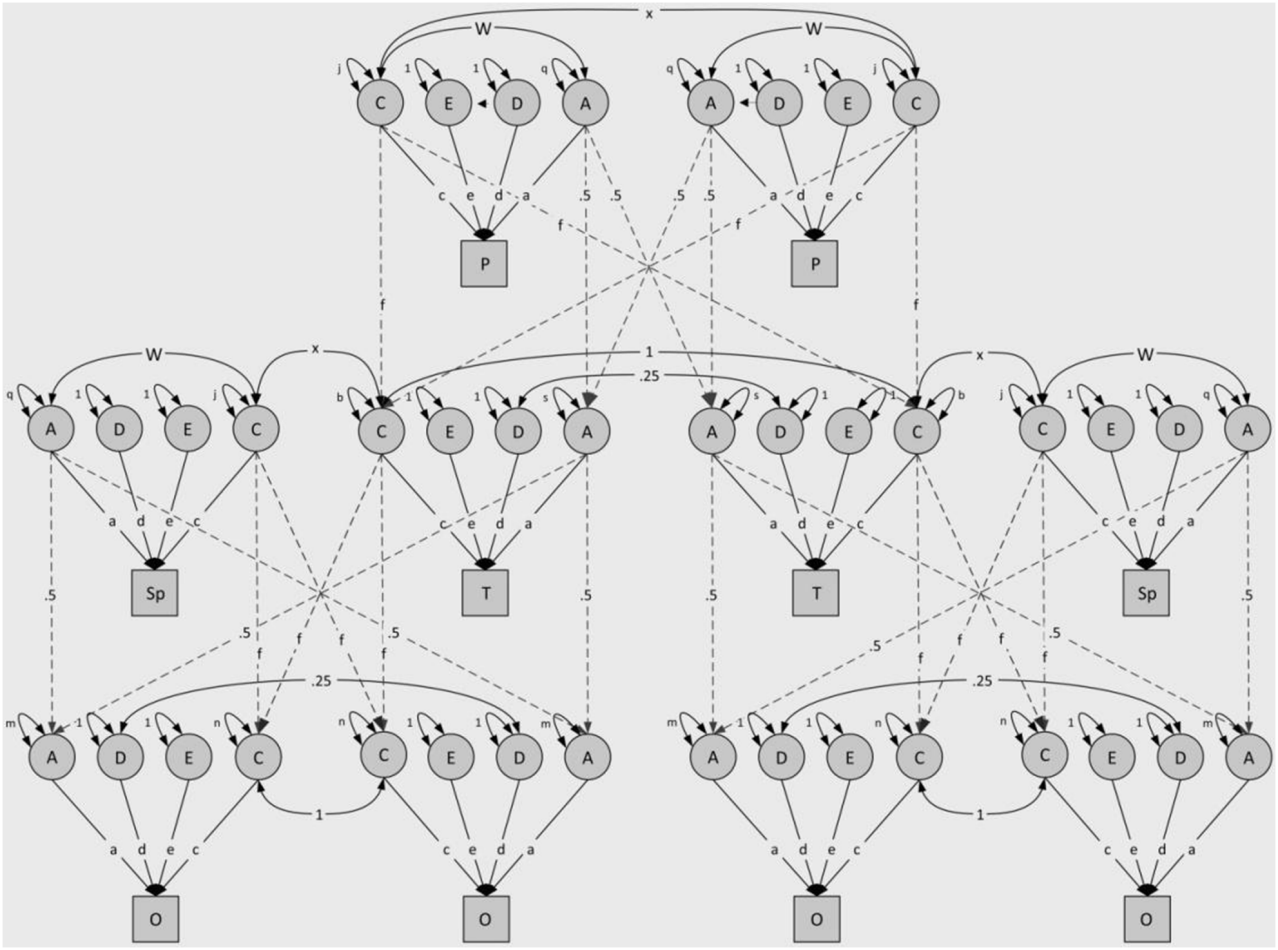
Genetic model for a DZ twin pair with parents, spouses and offspring. Notes: A = additive genetic effects, D = genetic dominance, E = non-shared environmental effects, C = shared environmental effects, f = cultural transmission path, w = gene-environment correlation, q = variance additive genetic effects, s = residual variance additive genetic effects twin generation, m = residual variance additive genetic effects offspring generation, j = variance shared environmental effects, b = residual variance shared environmental effects twin generation, n = residual variance additive genetic effects offspring generation, P = parent, T = DZ twin, Sp = spouse, O = offspring. Please note that additional siblings (and their spouses and offspring) are not included in the figure for reasons of convenience. Also note that only two children (per spouse pair) in the offspring generation are included in Figure 1 while a maximum of four is included in the analyses.

To assess genetic and environmental influences, we estimated 15 correlations between relatives in a saturated model that does not hold any assumptions regarding genetic and environmental influences underlying trust: MZ twins; DZ twins/sibling; parent-offspring; cousins avuncular via MZ twins; cousins avuncular via DZ twins/siblings; nieces/nephews via MZ twins; nieces/nephews via DZ twins/siblings; spouses; spouses in law via MZ twins; spouses in law via DZ twins/siblings; spouse-spouse via MZ twins; spouse-spouse via DZ/siblings; spouse in law avuncular via MZ twins; spouse in law avuncular via DZ twins/siblings; parent-offspring in law (see Figure 1). The pattern of phenotypic resemblance between pairs of relatives that differ in genetic and environmental similarity provides insight into the relative contribution of genetic and environmental influences. If a trait is under genetic influence, then relatives that are genetically more alike are expected to show a higher phenotypic resemblance.

In a twin-family design, the observed MZ correlation for a trait represents the upper boundary of the heritability. Correlations among other types of relatives, relative to the MZ correlation and relative to correlations among other relatives, help to quantify the heritability of the trait. We include many different genetic relations in our sample to precisely estimate different sources of variation. To illustrate, MZ twins are genetically 100% similar, DZ twins share on average 50% of their segregating genes, and cousins via DZ twins (CODZ) share on average 12.5% of their genes and spouses (SP) do not share any of their segregating genes. If a trait is heritable, then we expect phenotypic correlations to be a function of the degree of genetic resemblance. In this example, we would expect the phenotypic correlation pattern for a heritable trait to reflect rMZ > rDZ > rCODZ > rSP. However, if phenotypic correlations are equal between pairs of varying genetic similarity, then the trait is not under genetic influence. If we allow for more complex models, for example non-random mating where spouses select each other based on phenotype, differential correlational patterns are expected.

Subsequently, structural equation models were specified in which individual differences in trust were modeled as a function of genes and environment and possible effects of non-random mating of spouses. Two types of genetic influences (additive genetic factors (A) and genetic dominance (D)) and three types of environmental influences (shared environmental factors (C), cultural transmission (CT) and non-shared environmental factors (E)) were distinguished. ‘A’ represents additive effects of alleles summed over all loci. ‘D’ represents the extent to which the effects of alleles are not additive (genetic dominance or epistasis). ‘C’ represents common environmental influences that render offspring of the same family more alike. ‘CT’ represents shared environmental factors due to cultural transmission.

We should also briefly comment on the meaning of cultural transmission in the present model. Cultural transmission is the transmission from parents to their children of the environmental factors that are related to the traits under study (i.e., trust-in-others, and trust-in-self). In the present model, these environmental factors are transmitted from the parental environment to the offspring’s environment. Because children who are raised in the same home, grow up within a common environment as created by their parents, cultural transmission is by definition part of the shared environment in the offspring.

Presence of both cultural transmission and genetic transmission will result in a correlation between A and CT (i.e., rGE). ‘E’ represents all environmental influences that result in differences between members of a family. E also includes measurement error. Statistical significance of parameters was tested by comparing the fit of nested (increasingly more restricted) models to the fit of less restricted models, using maximum likelihood optimization, implemented in Mx software [21], using a criterion level α of .05 for all tests.

**Figure 2:**
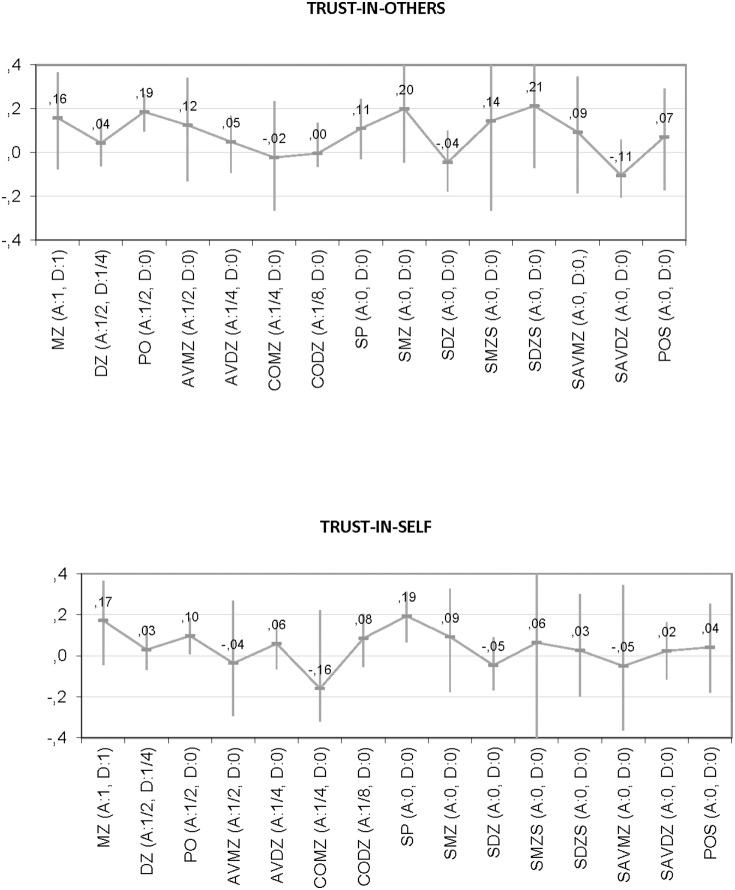
Weighted mean correlation (95% confidence interval) between relatives grouped by degree of genetic similarity for Trust-in-Others (top) and Trust-in-Self (bottom). Notes: correlations are constrained to be equal across twins and regular siblings and across sex; MZ = twin-twin MZ; DZ = twin-twin DZ/sibling; PO = parent-offspring; AVMZ = cousins avuncular through MZ; AVDZ = cousins avuncular through DZ/sibling; COMZ = niece/nephews through MZ; CODZ = niece/nephews through DZ/sibling; SP = spouse-pairs; SMZ = sister/brother in law through MZ; SDZ = sister/brother in law through DZ/sibling; SMZS = spouse-spouse through MZ; SDZS = spouse-spouse through DZ/sibling; SAVMZ = aunt/uncle cousin in law through MZ; SAVDZ = aunt/uncle cousin in law through DZ/sibling; POS = parent-offspring in law. The additive genetic correlation (A) and the non-additive genetic correlation (D) between two members of a relationship are within parentheses. For every phenotypic correlation the theoretical correlation for additive genetic influences (A) and non-additive genetic influences (D) is provided under the assumption of random mating. These correlations indicate the genetic resemblance between the different pairs of relatives.

Observed correlations between relatives in the saturated model were generally low and did not differ for pairs of relatives that differ in genetic relatedness (trust-in-others: χ^2^(14) = 18.31; *p* = .193; trust-in-self: χ^2^(14) = 10.40; *p* = .733), implying no significant genetic influences. They were also not different for pairs of relatives that were raised in the same family or not, suggesting no effect of shared environmental factors on trust. Figure 2 shows the observed correlations (and 95% confidence intervals) for pairs of relatives grouped by genetic relatedness for both trust-in-others and trust-in-self.

Table 2 lists the statistical properties of all models that were fitted to the data, including tests of significance (χ^2^ difference test) for all estimated parameters and additional statistics describing the fit of the different models (i.e., Akaike’s Information Criterion (AIC), Bayesian Information Criterion (BIC), and Deviance Information Criterion (DIC)).

**Table 2:** Model fitting results for Trust-in-Others (upper part) and Trust-in-Self (lower part) within an extended twin-family design.

	Model	Tested against	−2LL	df	par	cs	?^2^	Δdf	p-value	AIC	BIC	DIC
Trust-in-others												
O-1	saturated		5335.728	970	18	5				3395.73	−30.93	860.442
O-2	full genetic model	O-1	5344.67	976	11	4	8.94	6	.177	3392.67	−43.15	853.73
O-3	no D	O-2	5344.77	977	10	4	.10	1	.758	3390.77	−45.89	851.92
O-4	no D, A, r(GE)	O-2	5344.89	978	9	4	.22	2	.895	3388.89	−48.61	850.12
**O-5**	**no D, A, r(GE),C**	**O-2**	**5344.92**	**979**	**8**	**4**	**.25**	**3**	**.969**	**3386.92**	−**51.37**	**848.27**
O-6	no D, A, r(GE),C,CT	O-2	5361.22	980	7	4	16.55	4	.002	3401.22	−46.01	854.55
O-7	no D, CT,r(GE)	O-2	5348.983	978	9	4	4.31	2	.116	3392.983	−46.559	852.163
O-8	no D, CT,r(GE),C	O-2	5348.983	979	8	4	4.31	3	.230	3390.983	−49.341	850.299
Trust-in-self												
S-1	saturated		4841.874	981	18	5						
S-2	full genetic model	S-1	4845.47	987	11	4	3.59	6	.613	2869.72	−326.01	581.90
S-3	no D	S-2	4845.72	988	10	4	.26	1	.593	2868.51	−328.40	580.43
S-4	no D, A, r(GE)	S-2	4846.51	989	9	4	1.04	2	.601	2867.33	−330.77	578.98
S-5	no D, A, r(GE),C	S-2	4847.33	990	8	4	1.86	3	.141	2870.38	−331.03	579.64
**S-6**	**no D, A, r(GE),C,CT**	**S-2**	**4852.38**	**991**	**7**	**4**	**6.91**	**4**	**.613**	**2869.72**	−**326.01**	**581.90**

Notes: *−*2LL = minus 2 log likelihood; par = number of estimated parameters; cs = number of constraints in the model, χ2 = Chi square (difference in *−*2LL); Δdf = difference in degrees of freedom; p = p-value; AIC = Akaike’s Information Criterion; BIC = Bayesian Information Criterion; DIC = Deviance Information Criterion; O = others; S = self; r(GE) refers to the correlation between A and CT, if CT is eliminated from the model, r(GE) has to be fixed to zero as well; **if A is eliminated from the model, D and r(GE) will be estimated at zero as well. Models in which the effects of D are estimated but the effects of A are fixed to zero are biologically implausible [16], preferred models are printed in bold font.

For the trust-in-others, the decomposition of the variance into genetic and environmental components showed good fit to the data (Model O-2 in Table 2, χ^2^(6)  = 8.94; *ns*;). Within the full genetic model for trust-in-others, non-shared environmental factors explained 90% of the variance; the remaining variance was attributed to genetic dominance deviation (5%), and effects of cultural transmission (5%). Structural equation modeling showed that eliminating genetic effects or the effects of cultural transmission from the model did not result in a significant worsening of the model fit (Models O-3, O-4, O-5, O-7, and O-8 in Table 2), implying that these factors individually did not explain a significant part of the variance. A model in which all factors but non-shared environmental factors were eliminated, however, resulted in a significant deterioration of the model fit (Model O-6 in Table 2). This implies that 5% of the variance in trust-in-others is explained by either genetic or cultural transmission from parents to offspring Based on AIC, BIC, and DIC indices, a model in which the variance of trust-in-others is explained by non-shared environmental factors and effects of cultural transmission explains the data better than a model including non-shared environmental factors and additive genetic effects (Models O-5 and O-8 in Table 2, respectively).

Decomposition of the variance of the trust-in-self into genetic and environmental factors revealed good fit to the data (Model S-2 in Table 2: χ^2^(6)  = 3.59; *ns*). Within the full variance decomposition model, variance of trust-in-self is explained by non-shared environmental factors (86%), genetic dominance deviation (10%), additive genetic effects (3%), and effects of cultural transmission (1%). Genetic factors and effects of cultural transmission were however not significant (Model S-6 in Table 2).

The most parsimonious models were a model including E and CT for the trust-in-others scale in which E explained 95% of the variance and CT explained the remaining 5% of the variance. For the trust-in-self scale, the most parsimonious model included solely the E component; that is, 100% of the variance is explained by E. Based on the full model, broad sense heritability estimates for trust-in-others and trust-in-self were 5% and 13%, respectively, and not significantly different from zero (see Table 2). It could be argued that the lack of a significant heritability is due to a lack of power, even though the power is optimized due to the inclusion of many different pairs of relatives. However, the point estimates of heritability in the full model are quite modest, and the pattern of low correlations across all pairs of relatives are not suggestive of high heritability. Nevertheless, replication of the present results would provide more conclusive evidence for the absence of substantial genetic effects for trust. Because it was not feasible to examine the test-retest reliabilities in the present sample, we examined it in an independent sample (*n* = 59) addressing stability over a period of two months. Test-retest reliability for both scales were moderate or high (*r* = .76 and *r* = .53, respectively, see Methods and Materials), indicating that measurement error could not explain the high influence of non-shared environmental influences.

Finally, we have also conducted a series of analyses to investigate the impact of non-random mating on the variance decomposition of the trust-in-others and trust-in-self scales. Although the spousal correlations were moderately high and similar in magnitude as the MZ correlations (*r* = .10, *p* = .11; and *r* = .19, *p* = .004), for Trust-In-Others and Trust-In-Self, respectively), the associations did not affect the genetic variance in the sample under study. Genetic variance in the population will only be increased if there is genetic variance for the trait on which assortment is based. For both Trust-in-Others and Trust-in-Self, we do not observe genetic variance; hence, no increased genetic variance was observed. We have modeled the non-random mating as a function of the environment; that is, partners were believed to be alike because they share a similar environment (for a graphical representation, see Figure 1).

## Discussion

The present findings show that that the heritability of trust (trust-in-others, *h^2^* = 5%, *ns*; others’ trust-in-self, *h^2^* = 13%, *ns*) is virtually absent, and more modest than that of various expressions of abilities, psychological disorders, or classic personality traits. Clearly, such findings demonstrate an exception to the law that all human behavioral traits are quite heritable. Indeed, non-shared environmental influences accounted for virtually all of the phenotypic variation in our trust in others and our beliefs of others’ trust in ourselves.

We would like to point out that the estimates of the genetic and environmental parameters would have been different if only MZ and DZ twin pairs were considered in the analyses, i.e. as in the classical twin study. The rationale for estimating heritability in an extended twin-family design is that when estimating heritability based solely on MZ and DZ twin correlations, a number of significant assumptions have to be made. In a scenario where these assumptions do not hold, estimates of heritability may be biased. Estimating heritability in an extended twin-family design, as we did in the present study, allows testing some of the assumptions and therefore significantly reduces potential bias of the estimate of heritability. For example, in the classical twin design, where only the resemblance between MZ twins and between DZ twins is considered, we have to assume the absence of assortative mating, the absence of cultural transmission, and the absence of either non-additive genetic influences or shared environmental influences since these cannot be estimated at the same time. This is why we and others [22]–[23] have advocated previously that estimates from twin studies do not always correctly reflect the genetic and environmental sources of co-variation.

If the current study had been based on solely MZ and DZ twins, we would have concluded that the heritabilities of trust-in-others and trust-in-self are .16 and .17, respectively and that all genetic variance would be non-additive. Note that when the DZ correlation is less than .5 of the MZ correlation, as in the present research for both trust-scales, the heritability as calculated from twin correlations typically equals the MZ correlation [24]. Information obtained from including pairs of relatives other than MZ and DZ twins in the study clearly shows that this conclusion would have been incorrect. For both trust-in-others and trust-in-self we observe phenotypic resemblance for pairs of relatives with no non-additive genetic similarity, which does not agree with the conclusion that all genetic variance is non-additive. Moreover, the pattern of phenotypic correlations that we observe when considering all pairs of relatives does not follow a pattern of genetic resemblance (i.e., phenotypic correlations do not follow the decline in genetic similarity between more distant relatives) indicating that genetic influences are modest at most (i.e. 16–17%) yet probably absent.

The low impact of genetic influences on trust is remarkable compared to heritability estimates for other traits such as autism (*h^2^* = 90%) [25], schizophrenia, (*h^2^* = 81%) [26], major depression (*h^2^* = 37%) [27], or general anxiety (*h^2^* = 32%) [28], or general cognitive ability (*h^2^* = 80%) [17]. What is perhaps even more remarkable is that in the same sample, the heritability estimates were a fair amount higher for childhood experiences, such as reading experiences and family functioning (*h^2^* = 62%), leisure time activities (*h^2^* = 52%), social network (*h^2^* = 36%), and even life events (*h^2^* = 29%) [18]. As such, beliefs about human nature, as reflected by trust-in-others, and other people’s beliefs in themselves, seem to be strongly shaped by non-genetic influences. We know of only one published study among a specific sample of adolescents and young adults (17–33 years) that revealed for only some judgments that are linked to trust a heritability greater than 20%, but this was not observed for a negative item, “people take advantage” which yielded a heritability of 14% [29]. And a study on behavioral trust in a trust game revealed a heritability of 10% to 20% [30], but behavioral trust may also assess risk-taking, cooperative motivation, and may not be temporally stable; also, this particular study included a modest sample size (329 same sex twins, including 71 DZ and 258 MZ twins). In contrast, the heritability of giving and risk-taking tend to be in the 20%–30% range [31], as are various political attitudes and beliefs that vary on the conservative-liberalism spectrum [32]. Hence, the genetics of politics, and giving, is likely to be accounted for by variables other than trust, such as flexibility in information processing [33] or values regarding solidarity and egalitarianism [34].

The present findings suggest the importance of distinguishing between individual differences that are prominently shaped by others in our social interactions – as cause *and* consequence – and individual differences that are more strongly shaped by the self in social interactions, such as the classic variables of extraversion and agreeableness [2] as well as giving and risk-taking [31]. Trust, in particular, might be strongly shaped by the persons we encounter, and less so by the self. Moreover, in light of the variety of interaction partners and situations that different people encounter, it is understandable that the genetic influences for trust indeed tend to be relatively modest. Most of the variance is accounted for by non-shared environmental influences. We should note that the various kinds of gene-environment interactions may be included in our estimate of the non-shared environmental influences, and so perhaps some genetic effects on trust do exist and are revealed through gene-environment interactions. A case in point is when people differ in terms of having direct or indirect experiences with social exclusion, burglary or assault, which in combination with a trust-relevant genetic background may facilitate the expression of low trust. This may happen, for example, when people (based on their genetic make-up) are more likely than others to be involved in situations or exposed to negative views of humankind (e.g., people who professionally deal with detecting fraud and crime versus those who professionally are more likely to witness the goodness of people) that might undermine or strengthen our feelings and beliefs relevant to trust.

But in the absence of strong genetic influences, what *does* account for differences in trust? Note that the differences between families did not seem to matter much. The variance accounted for by cultural transmission was 5% for trust-in-others, and even non-significant for trust-in-self. Hence, social interaction experiences, direct or vicarious, outside of the immediate family are perhaps more essential than often is assumed. Beliefs relevant to trust may well be shaped by social interactions at school or at work, as well as by some observations of interactions in the various media, where trust-supporting and trust-undermining experiences take place [35]–[36].

Indeed, it is plausible that to some degree there are no strong genetic influences on the likelihood of being involved in situations that might strongly undermine or strengthen trust. For example, it is possible that specific yet powerful experiences (such as being fired or victim of burglary) or some societal trend (such as economic decline) help us understand variation in trust that would seem stable over at least some time. It does not need to be stable “personalities” per se that is captured by our measurements of trust-in-others and trust-in-self. One might speculate that at least in part the measurements capture a change in beliefs and orientation that will last for some time, but that may over longer periods of time return to those aspects of the self (base-line levels) that we tend to call personality. And it is possible that the base-line levels themselves are subject to systematic change. The present findings reveal a tendency that that trust may decline with age, which is interesting in light of previous evidence demonstrating cooperative and prosocial orientation increases with age [37]. People may become more prosocial in orientation despite a modest decline in trust.

## Conclusions

One of the most “social” traits of all – trust-in-others, trust-in-self – seems to challenge the first law of behavior genetics: Perhaps everything is heritable, but trust is only modestly heritable, at best. At the same time, given that we did not find any effect of shared environment, the second law of behavior genetics seems supported: The effect of being raised in the same family is generally smaller than the effect of genes. The challenge for future research is, therefore, to uncover the role of interaction experiences, and perhaps (social) media experiences, outside of the family that might help shape the trust we have in others, and the trust we think other people have in us.
